# Genetic Issues in the Diagnosis of Dystonias

**DOI:** 10.3389/fneur.2013.00034

**Published:** 2013-04-10

**Authors:** Simona Petrucci, Enza Maria Valente

**Affiliations:** ^1^Neurogenetics Unit, CSS-Mendel Laboratory, IRCCS Casa Sollievo della SofferenzaSan Giovanni Rotondo, Italy; ^2^Department of Experimental Medicine, “Sapienza” University of RomeRome, Italy; ^3^Department of Medicine and Surgery, University of SalernoSalerno, Italy

**Keywords:** dystonia, genetic, diagnosis, DYT1, DYT6, non-motor, phenotype, counseling

## Abstract

Dystonias are heterogeneous hyperkinetic movement disorders characterized by involuntary muscle contractions which result in twisting and repetitive movements and abnormal postures. Several causative genes have been identified, but their genetic bases still remain elusive. Primary Torsion Dystonias (PTDs), in which dystonia is the only clinical sign, can be inherited in a monogenic fashion, and many genes and loci have been identified for autosomal dominant (DYT1/*TOR1A*; DYT6/*THAP1*; DYT4*/TUBB4a*; DYT7; DYT13; DYT21; DYT23/*CIZ1*; DYT24/*ANO3*; DYT25/*GNAL*) and recessive (DYT2; DYT17) forms. However most sporadic cases, especially those with late-onset, are likely multifactorial, with genetic and environmental factors interplaying to reach a threshold of disease. At present, genetic counseling of dystonia patients remains a difficult task. Recently non-motor clinical findings in dystonias, new highlights in the pathophysiology of the disease, and the availability of high-throughput genome-wide techniques are proving useful tools to better understand the complexity of PTD genetics. We briefly review the genetic basis of the most common forms of hereditary PTDs, and discuss relevant issues related to molecular diagnosis and genetic counseling.

## Introduction

Dystonias are a heterogeneous group of hyperkinetic movement disorders characterized by involuntary muscle contractions, resulting in twisting and repetitive movements and abnormal postures (Fahn, 1988). Although the diagnosis is mainly clinical (Albanese and Lalli, 2012), the current classification is based on etiology, distinguishing primary torsion dystonias (PTDs), dystonia-plus syndromes, heredodegenerative disorders and secondary dystonias (Bressman, 2004). With the exclusion of secondary forms which are related to acquired causes, dystonic syndromes have a genetic basis. Several genes and loci have been identified (named with the conventional DYT symbol followed by progressive numbers – see Table 1), yet a genetic diagnosis remains elusive in the majority of patients. This review will focus on PTDs, tackling current achievements, and challenges in genetic diagnosis.

**Table 1:** **Molecular classification of “DYT” dystonia syndromes**.

Disease (MIM)	Gene	Locus	Phenotype	Inheritance
**PURE PRIMARY TORSION DYSTONIA**				
DYT1 (128100)	*TOR1A*	9q34	Early-onset generalized limb onset dystonia	AD
DYT2 (224500)	–	–	Early-onset generalized dystonia with prominent cranio-cervical involvement	AR
DYT4 (128101)	*TUBB4a*	19p13.12–13	Whispering dysphonia	AD
DYT6 (602629)	*THAP1*	8p11.21	Generalized cervical and upper-limb-onset dystonia	AD
DYT7 (602124)*	–	18p	Adult-onset cervical dystonia	AD
DYT13 (607671)	–	1p36.32–p36.13	Cervical and upper-limb dystonia	AD
DYT17 (612406)	–	20p11.2–q13.12	Segmental or generalized dystonia with prominent dysphonia	AR
DYT21 (614588)	–	2q14.3–q21.3	Adult-onset generalized or multifocal dystonia, often starting with blepharospasm	AD
DYT23 (614860)	*CIZ1*	9q34	Adult-onset cervical dystonia	AD
DYT24 (615034)	*ANO3*	11p14.2	Cranio-cervical dystonia with laryngeal and upper-limb involvement	AD
DYT25 (615073)	*GNAL*	18p11	Adult-onset cervical dystonia	AD
**PRIMARY DYSTONIA-PLUS SYNDROME**				
DYT5 (218230)	*GCH1*	14q22.2	Dopa-responsive dystonia	AD
THD (605407)	*TH*	11p15.5	Dopa-responsive dystonia	AR
DYT11 (159900)	*SGCE*	7q21.3	Myoclonus-dystonia	AD
DYT12 (128235)	*ATP1A3*	19q13.2	Rapid-onset dystonia parkinsonism	AD
DYT15 (607488)	–	18p11	Myoclonus-dystonia	AD
DYT16 (612067)	*PRKRA*	2q31.2	Early-onset dystonia parkinsonism	AR
**PAROXYSMAL SYNDROME**				
DYT8 (118800)	*MR1*	2q35	Paroxysmal non-kinesigenic dyskinesia (PNKD)	AD
DYT9 (601042)/DYT18 (612126)	*SLC2A1*	1p34.2	Paroxysmal dyskinesias with episodic ataxia and spasticity/paroxysmal exercise-induced dystonia (PED)	AD
DYT10 (128200)	*PRRT2*	16p11.2	Paroxysmal kinesigenic dyskinesia (PKD)	AD
DYT19 (611031)	–	16q13–q22.1	Paroxysmal kinesigenic dyskinesia 2 (PKD2)	AD
DYT20 (611147)	–	2q31	Paroxysmal non-kinesigenic dyskinesia 2 (PNKD2)	AD
**HEREDODEGENERATIVE DYSTONIA SYNDROME**				
DYT3 (314250)	TAF1	Xq13.1	Dystonia parkinsonism	X-R

AD, autosomal dominant; AR autosomal recessive: X-R, X linked recessive. *DYT7 locus on chromosome 18p has been recently questioned (Winter et al., 2012).

## The COBWEB of PTDs

In PTDs, dystonia is the only clinical sign (apart of tremor), without evidence of identifiable exogenous causes or neurodegeneration. PTDs represent the third most common movement disorder after Parkinson’s disease (PD) and essential tremor, and include about 75% of dystonia cases (Phukan et al., 2011). The clinical spectrum is broad and there is often a strong relationship between age at onset and clinical severity. In early-onset PTDs, dystonia usually generalizes within 5–10 years while, in late-onset forms, dystonia tends to remain focal or segmental (Ozelius and Bressman, 2011). A substantial proportion of early-onset cases (<26 years) have a monogenic basis with autosomal dominant (AD) or, more rarely, recessive (AR) inheritance (Table 1). Conversely, late-onset PTDs are complex disorders in which genetic and environmental risk factors variably interplay to determine the phenotype (Defazio et al., 2007). Yet this paradigm does not always hold true in atypical cases where blurred clinical signs and overlapping phenotypes can make genetic diagnosis a difficult task.

### Autosomal dominant PTD

DYT1-dystonia is the most common form of early-onset PTD, with an estimated frequency of 1/9,000 in Ashkenazi Jewish population and 1/160,000 worldwide (Bressman et al., 2000). A recurrent 3 bp deletion (delGAG) in the *TOR1A* (TorsinA) gene on chromosome 9q34.11 is found in nearly all mutated cases (Ozelius et al., 1997). The delGAG results in a glutamic acid residue loss in the C-terminus conserved region of TorsinA protein; mutant TorsinA shows aberrant cellular localization and impaired interaction with other proteins, resulting in stress-induced abnormalities, synaptic vesicle recycling defects, and altered development of neuronal pathways (Granata and Warner, 2010).

This mutation is found in diverse ethnicities, either inherited as a founder mutation or – *de novo* (Klein et al., 1998; Ozelius and Bressman, 2011), and only two additional mutations of unclear pathogenicity (p.R288Q and p.F205I) have been described in isolated atypical cases (Zirn et al., 2008; Calakos et al., 2010). The delGAG is inherited as an AD trait and only 20–30% of mutation carriers develop the disease (incomplete penetrance); interestingly, penetrance appears to be markedly lower (∼3%) in patients carrying the p.D216H polymorphism (rs1801968), suggesting that this variant could act as a genetic modifier of the delGAG mutation (Kamm et al., 2008). In most cases DYT1-dystonia starts during childhood in a limb, with generalization occurring within a few years from onset, but usually sparing the cranial-cervical region. More rarely, dystonia may remain segmental or purely focal (Ozelius and Bressman, 2011). Overall, clinical manifestations can vary widely even within families, ranging from dystonic storm to mild writer’s cramp (Opal et al., 2002; Edwards et al., 2003).

DYT6-dystonia is also a pure AD PTD, with reduced penetrance (60%) and variable expressivity (Saunders-Pullman et al., 2007). DYT6-dystonia is characterized by juvenile onset and frequent generalization but, in contrast to DYT1-dystonia, cranial-cervical, and laryngeal areas are frequently involved as the starting site. Nevertheless, onset in adult age or in a limb, and lack of generalization, have all been reported (LeDoux et al., 2012). Mutations in the *THAP1* (thanatos associated protein-1) gene on chromosome 8p21–p22 were initially identified in Amish–Mennonite families (Fuchs et al., 2009). Subsequently, about 60 familial and sporadic cases have been reported worldwide (Blanchard et al., 2011). Despite its dominant inheritance, a homozygous mutation in *THAP1* has been described in a consanguineous family with generalized dystonia (Schneider et al., 2011). Since almost each case bears a unique mutation, molecular diagnosis requires direct sequencing of the whole *THAP1* coding region.

Recently, thanks to the advent of Whole Exome Sequencing (WES) technology, four new genes have been implicated as causative of AD focal/segmental dystonia. In one large Australian family with fully penetrant juvenile-adult-onset “whispering dysphonia,” occasional generalization and alcohol benefit (Wilcox et al., 2011), the p.R2G mutation in the *TUBB4a* gene (DYT4, encoding the isoform a of b-Tubulin) has been identified by exome sequencing (Hersheson et al., 2012). Molecular screening of 394 phenotypically similar patients identified a novel mutation (p.A271T) in one case (Lohmann et al., 2013). Mutations in *CIZ1* (DYT23, *CDKN1A-*interacting zinc finger protein-1), *ANO3* (DYT24, Anoctamin 3, encoding a calcium-gated chloride channel highly expressed in the striatum), and *GNAL* (DYT25, guanine nucleotide-binding protein, alpha-activating activity polypeptide, olfactory type), have been identified in families with juvenile-adult-onset cervical or cranial-cervical dystonia and in a few sporadic cases with similar phenotypes (Charlesworth et al., 2012; Xiao et al., 2012; Fuchs et al., 2013). In particular, *GNAL* mutations were detected in additional 6 out of 39 families (15%), suggesting they could represent a major cause of adult-onset cranial-cervical dystonia PTD.

Other AD PTD loci have been described in isolated familial cases. The DYT7 locus was mapped to chromosome 18p in a family with cervical PTD (Leube et al., 1996), but its existence has been recently questioned (Winter et al., 2012). The DYT13 locus was mapped to chromosome 1p36 in an Italian family with juvenile onset dystonia, prominent cranial-cervical and arm involvement, and occasional generalization (Bentivoglio et al., 2004) while, in another family with adult-onset generalized/multifocal dystonia, often starting with blepharospasm, the DYT21 locus was mapped to 2q14.3–q21.3 (Norgren et al., 2011). For both loci, the causative gene is still missing.

### Autosomal recessive PTD

Few families with AR PTD have been described to date. These include DYT2, an early-onset PTD with limb involvement and rapid generalization (Khan et al., 2003), and DYT17, a juvenile segmental/generalized dystonia with severe dysphonia mapped to chromosome 20p11.2–q13.12 in a Lebanese family (Chouery et al., 2008). The causative genes for these Mendelian PTDs still have to be identified and their detection represents a challenge to address with innovative techniques such as WES.

### Sporadic late-onset PTD

Late-onset PTD is about 10 times more frequent than early-onset PTD with an estimated prevalence of 30/100,000 worldwide [Epidemiological Study of Dystonia in Europe (ESDE) Collaborative Group, 2000]. The disease usually begins in adulthood and shows limited tendency to spread or generalize. The majority of late-onset PTD are sporadic, yet a positive family history is present in up to 25% of first-degree relatives, supporting the existence of genetic factors that could influence PTD susceptibility (Defazio et al., 2012). It is hypothesized that most cases have a multifactorial basis, in which common susceptibility genes interplay with individual genetic and environmental risk factors to reach a threshold of disease (Defazio et al., 2007).

In order to show an association between specific genetic variants and focal dystonias, many case-control studies have been performed, focusing on candidate genes such as those related to dopaminergic transmission, brain plasticity, and genes causative of Mendelian PTDs; overall, these studies have yielded contradictory outcomes. The (CT/GT/GA)_n_ polymorphism in the dopamine D5 receptor gene has been associated with adult-onset focal dystonia in patients of European ancestry, but not in other cohorts. Similarly, a functional polymorphism (p.V66M, rs6265) in the brain-derived neurotrophic factor (BDNF) gene and variants in the *THAP1* gene have been associated to PTD risk, but with inconsistent results. More extensive work has been performed on *TOR1A* variants. The p.D216H SNP was reported as a risk factor in patients with familial focal/segmental dystonia, but these results were not replicated in other studies. Several SNPs from the 3′ untranslated region of *TOR1A* have also been implicated in focal dystonia as predisposing factors in some studies but protective in others. These contradictory data are likely due to confounding factors including the small number of SNPs tested, limited samples size, clinical heterogeneity of PTDs and different ethnicity (Ozelius and Bressman, 2011).

## New Approaches to Improve Knowledge on PTD Genetics

Although 15 years have passed from the discovery of the first PTD gene (Ozelius et al., 1997), the identification of new dystonia genes has progressed slowly. This relates to the scarcity of large families with several affected members, the fragmentary knowledge about the pathogenetic mechanisms of dystonias and the difficulty in identifying clinically homogeneous families or groups of patients for genetic studies. Recently, the identification of PTD endophenotypes, the characterization of interactions between dystonia-related proteins and the advent of innovative techniques such as WES and genome-wide association studies (GWAS), provide new hope in clarifying aspects of PTD.

### Unraveling PTD endophenotypes

A major limitation in assessing PTD families is that clinical investigation is usually restricted to the motor phenotype, although non-motor features have also been detected (Stamelou et al., 2012). Recently, several studies based on neuroimaging, neurophysiological, and expression profiling approaches have reported subclinical abnormalities (endophenotypes) in healthy DYT1/DYT6 mutation carriers (Fiorio et al., 2007; Bradley et al., 2009; Walter et al., 2010; Niethammer et al., 2011; Phukan et al., 2011). For instance, magnetic resonance diffusion tensor imaging used in conjunction with probabilistic tractography have demonstrated an anatomical disruption of cerebellar outflow in all DYT1/DYT6 carriers regardless of the phenotype, while a second protective downstream alteration of thalamo-cortical projections was found in non-manifesting carriers only (Argyelan et al., 2009). FDG-PET studies have shown relative metabolic increases in presupplementary motor area and parietal association regions in DYT1/DYT6 patients and a different pattern of abnormalities in non-manifesting carriers (Carbon et al., 2004). In Raclopride-PET imaging studies, significant reductions in radioligand binding have been detected in both DYT1 and DYT6 carriers, being greater in the latter, suggesting an intriguing link between DYT6 and dopamine function (Carbon et al., 2009).

Similar approaches have been employed to detect endophenotypes among healthy relatives of patients with focal dystonias, whose genetic bases are largely unknown. For example, somatosensory spatial and temporal discrimination thresholds (TDT) have been found altered not only in focal dystonia patients (Sanger et al., 2001; Scontrini et al., 2009; Tinazzi et al., 2012) but also in some healthy family members. Interestingly in these subjects a correlation has been reported between abnormal tactile TDT and a bilateral increase in putaminal gray matter detected by Voxel Based Morphometry (Bradley et al., 2009).

Moreover, these findings may shed light on pathophysiological mechanisms underlying primary dystonias. It is now established that PTDs, traditionally regarded as a mere basal ganglia disease without structural abnormalities, might be considered a neurodevelopmental circuit disorder, in which maladaptive plasticity in the sensory-motor cortex (Quartarone et al., 2009) and impaired inhibition at different levels of the central nervous system are necessary to produce an overt motor manifestation (Albanese and Lalli, 2012). As it is now clear that non-motor features are part of the pathophysiological “fingerprint” of dystonia, endophenotypes could represent valuable tools for future research. In this light, neurophysiology and neuroimaging may facilitate the identification of clinically non-manifesting mutation carriers within PTD families, or may allow the clustering of homogeneous groups for genetic studies (Stamelou et al., 2012).

### Cues from PTD pathogenesis

The PTDs phenotypic variability suggests genetic heterogeneity. However clinical overlaps, not only within early-onset PTDs but also with the more common focal/segmental dystonias, suggest that common pathogenic mechanisms may be involved. Indeed, recent functional studies have demonstrated that *THAP1* and *TOR1A* interact in a common pathway.

TorsinA, a member of the AAA+ superfamily, is involved in multiple cellular functions (vesicle fusion, membrane trafficking, protein folding, and cytoskeletal dynamics) and has a protective role by maintaining a homeostatic threshold against the endothelial reticulum (ER) stress. When mutated, its buffering capacity is greatly diminished, its ATPase activity reduced and its ER translocation prevented (Granata and Warner, 2010). Thap1 is a nuclear transcription factor that can regulate endothelial cell proliferation (Cayrol et al., 2007) but its function in the brain is still unknown. *In vitro* studies have shown that Thap1 physically interacts with *TOR1A* and represses TorsinA expression by binding to the *TOR1A* promoter; this interaction is abolished by pathogenic mutations, leading to a decreased repression (Kaiser et al., 2010). However, the role of Thap1 as a major genetic modifier in DYT1-dystonia has been recently questioned (Palada et al., 2012). In line with this, Thap1 is predicted to regulate several other gene targets, including the *TAF1* gene which is also implicated in DYT3 dystonia (Mazars et al., 2010).

The identification of the novel dystonia genes are contributing to shed light on the pathogenetic mechanisms of PTD. *TUBB4a* is supposed to be involved in neuronal cytoskeletal defects (Hersheson et al., 2012), while *ANO3* and *GNAL* mutations point to a key role for striatal neurons in the pathophysiology of dystonia, either through abnormal neuronal excitability related to the malfunctioning of chloride channels or through abnormalities of dopamine and/or adenosine signal transduction pathways (Charlesworth et al., 2012; Fuchs et al., 2013). However functional studies are needed in order to assess their specific role in the pathophysiology of dystonia and their potential interactions with other PTD genes.

### Novel high-throughput strategies for identification of PTD-related genes

To date, the identification of PTD genes and genetic risk factors has proven to be a hard task. Recently, however, the introduction of novel high-throughput technologies (WES and GWAS) have boosted these fields (Kumar et al., 2012).

Whole exome sequencing can identify novel disease-causative genes by sequencing the entire human exome in a single experiment, and has truly revolutioned gene identification in Mendelian disorders. In the field of dystonias, the first result was seen in Paroxysmal Kinesigenic Dyskinesia (PKD, DYT10 locus), a rare AD episodic dystonic syndrome characterized by recurrent and brief attacks of involuntary movements triggered by sudden voluntary movements. Overlapping phenotypes include Infantile Convulsions and Paroxysmal Choreoathetosis (ICCA) and Benign Familial Infantile Seizures syndrome (BFIS). PKD, ICCA, and other related phenotypes had been mapped to distinct loci at the pericentromeric region of chromosome 16 (Tomita et al., 1999; Swoboda et al., 2000; Valente et al., 2000), but the causative gene(s) had long been elusive. Using WES, several groups independently identified mutations in the *PRRT2* gene as the major cause of PKD and ICCA syndrome (Chen et al., 2011; Wang et al., 2011; Lee et al., 2012), demonstrating the inaccuracy of mapping data. After gene identification, mutations were also identified in pure BFIS families (Ono et al., 2012), as well as in occasional cases with hemiplegic migraine (Dale et al., 2012). This confirmed that mutations in the same gene can cause heterogeneous phenotypes, suggesting a genetic complexity previously unexpected for Mendelian disorders (Schmidt et al., 2012). Subsequently, the same WES approach has been successfully used to identify four novel genes causative of late-onset PTD, as previously described.

Genome-wide association studies, on the other hand, aim at identifying polymorphic variants acting as risk factors for complex diseases via large-scale, population-based studies. Thousands of common variants (minor allele frequency >5%) can be simultaneously genotyped in large cohorts of patients and controls, followed by a stringent statistical analysis to discriminate real hits from false positive results. In movement disorders, this approach has been largely adapted to PD, although the number of significant hits has been unexpectedly low and only few genes, such as *SNCA*, *MAPT*, and *LRRK2*, have been found to harbor variants robustly associated with the disease. A recent meta-analysis of GWAS studies in PD identified 17 additional disease risk loci which surpassed the threshold for genome-wide significance, all with low odds ratios (Kumar et al., 2012). To date there are no published GWAS for PTDs, but their potential contribution to the field is so obvious that many such projects are currently in progress. It is expected that the new insights on PTD endophenotypes and the possibility to integrate worldwide data with a meta-analytic approach, are going to shrink some of the confounding factors that could have compromised previous association studies.

## Merging Past and Future: Genetic and Counseling in PTDs

**Figure 1:**
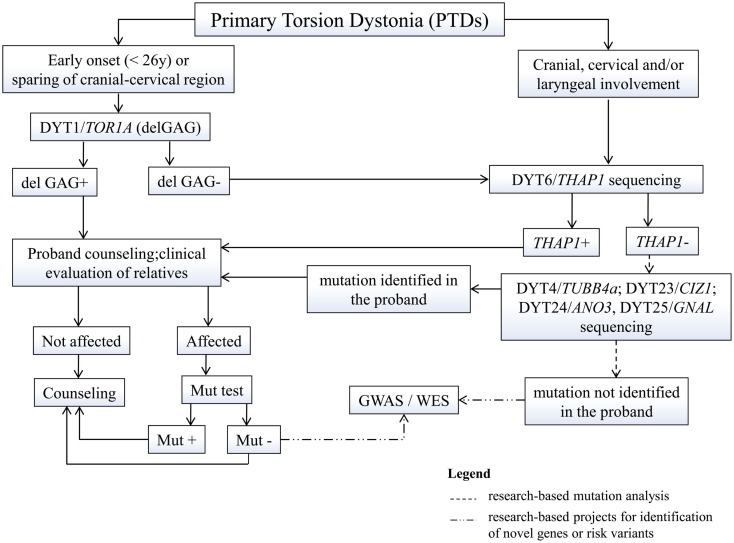
**Proposed flowchart for genetic testing in primary dystonias**. GWAS, genome-wide association studies; WES, whole exome sequencing; mut, mutation.

Diagnosis of dystonia is mainly clinical, yet genetic analysis and counseling is being requested more and more often. In primary dystonias, genetic testing is only available for those forms of early-onset PTD for which the gene is known (Figure 1). Testing of the DYT1-delGAG, that is readily available and relatively inexpensive, should be recommended for any PTD case with onset before 26 years and in those with later onset who have an affected relative with early-onset dystonia. Other *TOR1A* mutations are extremely rare and whole gene sequencing is not recommended in clinical practice (Albanese et al., 2011). Sequencing of the other known PTD genes (*THAP1*, *TUBB4a*, *CIZ1*, *ANO3*, and *GNAL*) should be considered in patients with predominant cranial-cervical and laryngeal involvement, especially if family history is positive, with prioritization according to the age at onset and distribution of dystonia.

The identification of mutations in a proband requires a detailed assessment of family history, given their dominant inheritance and variable expression. Once a mutation is identified in a proband, carrier testing is available for at-risk asymptomatic relatives who may request it. This type of testing should never be performed on minors and should always be accompanied by pre-test genetic counseling. Since most PTD mutations show reduced penetrance, it is impossible to predict whether a mutation carrier will develop the disease, although such probability will progressively decrease with age, especially for early-onset conditions such as DYT1 and DYT6. Yet, in case of positive testing, they need to understand the 50% risk to transmit the mutation to their offspring who could eventually develop the disease, based on estimated penetrance.

Genetic diagnosis in sporadic late-onset PTD is much more complex as several genetic and environmental factors are likely to be involved. GWAS are significantly improving our understanding of the complexity of sporadic PTDs, allowing the identification of genetic susceptibility or protective factors for the development of the disease. Yet, to avoid false expectation from commercially available genetic profiles, it should be remembered that such genetic factors are relevant only in large population studies, while they are of limited significance in personalized medicine (Kumar et al., 2012).

## Conflict of Interest Statement

Prof. Valente serves on the editorial boards of BMC Neurology, Movement Disorders, and Pediatric Research. She is the recipient of a European Research Council Starting Grant, and of research grants from the Italian Ministry of Health, the Italian Ministry of University and Research, the Telethon Foundation Italy and the European Community FP7 Program.
